# Factors Controlling Floc Formation and Structure in the Cyanobacterium *Synechocystis* sp. Strain PCC 6803

**DOI:** 10.1128/JB.00344-19

**Published:** 2019-09-06

**Authors:** Fabian D. Conradi, Rui-Qian Zhou, Sabrina Oeser, Nils Schuergers, Annegret Wilde, Conrad W. Mullineaux

**Affiliations:** a School of Biological and Chemical Sciences, Queen Mary University of London, London, United Kingdom; b Institute of Biology III, University of Freiburg, Freiburg, Germany; Rutgers University-Robert Wood Johnson Medical School

**Keywords:** type IV pili, biofilms, cell adhesion, cyanobacteria, cyanobacteriochrome, flocculation, photoreceptors

## Abstract

Motile strains of the unicellular cyanobacterium *Synechocystis* sp. strain PCC 6803 readily aggregate into flocs, or floating multicellular assemblages, when grown in liquid culture. As described here, we used confocal imaging to probe the structure of these flocs, and we developed a quantitative assay for floc formation based on fluorescence imaging of 6-well plates. The flocs are formed from strands of linked cells, sometimes packed into dense clusters but also containing voids with very few cells. Cells within the dense clusters show signs of nutrient stress, as judged by the subcellular distribution of green fluorescent protein (GFP)-tagged Vipp1 protein. We analyzed the effects on flocculation of a series of mutations that alter piliation and motility, including Δ*hfq*, Δ*pilB1*, Δ*pilT1*, and Δ*ushA* mutations and deletion mutations affecting major and minor pilins. The extent of flocculation is increased in the hyperpiliated Δ*pilT1* mutant, but active cycles of pilus extension and retraction are not required for flocculation. Deletion of PilA1, the major subunit of type IV pili, has no effect on flocculation; however, flocculation is lost in mutants lacking an operon coding for the minor pilins PilA9 to -11. Therefore, minor pilins appear crucial for flocculation. We show that flocculation is a tightly regulated process that is promoted by blue light perception by the cyanobacteriochrome Cph2. Floc formation also seems to be a highly cooperative process. A proportion of nonflocculating Δ*hfq* cells can be incorporated into wild-type flocs, but the presence of a high proportion of Δ*hfq* cells disrupts the large-scale architecture of the floc.

**IMPORTANCE** Some bacteria form flocs, which are multicellular floating assemblages of many thousands of cells. Flocs have been relatively little studied compared to surface-adherent biofilms, but flocculation could play many physiological roles, be a crucial factor in marine carbon burial, and enable more efficient biotechnological cell harvesting. We studied floc formation and architecture in the model cyanobacterium *Synechocystis* sp. strain PCC 6803, using mutants to identify specific cell surface structures required for floc formation. We show that floc formation is regulated by blue and green light perceived by the photoreceptor Cph2. The flocs have a characteristic structure based on strands of linked cells aggregating into dense clusters. Cells within the dense clusters show signs of nutrient stress, pointing to a disadvantage of floc formation.

## Abstract (precis)

Some bacteria form flocs, which are multicellular floating assemblages of many thousands of cells. Flocs have been relatively little studied compared to surface-adherent biofilms, but flocculation could play many physiological roles, be a crucial factor in marine carbon burial, and enable more efficient biotechnological cell harvesting. We studied floc formation and architecture in the model cyanobacterium *Synechocystis* sp. strain PCC 6803, using mutants to identify specific cell surface structures required for floc formation. We show that floc formation is regulated by blue and green light perceived by the photoreceptor Cph2. The flocs have a characteristic structure based on strands of linked cells aggregating into dense clusters. Cells within the dense clusters show signs of nutrient stress, pointing to a disadvantage of floc formation.

## INTRODUCTION

The unicellular motile freshwater cyanobacterium *Synechocystis* sp. strain PCC 6803 (hereinafter *Synechocystis*) is an important model organism for studies on photosynthesis. *Synechocystis* cells (and those of other cyanobacteria) do not possess flagella, instead performing twitching motility via type IV pili (T4P) (reviewed in reference 1). T4P are protein filaments that are extended and retracted from a membrane-spanning base using ATPase motor proteins (2, 3). The tip of the pilus attaches to surfaces, generating substantial force upon retraction (4, 5) and pulling the cell across the surface.

T4P and twitching motility have proven essential for biofilm initiation and substructure formation in the model heterotrophic bacterium Pseudomonas aeruginosa (6, 7). However, their role in biofilm formation in cyanobacteria remains under investigation. T4P are dispensable for biofilm formation in the nonmotile cyanobacterium Synechococcus elongatus PCC 7942 (8). However, deletion of *pilC*, encoding the inner membrane platform of the T4P system, was recently shown to significantly reduce biofilm formation and flocculation in *Synechocystis* (9).

In *Synechocystis*, the T4P filaments are mainly formed by PilA1 (10), an initially membrane-anchored major pilin that is polymerized to yield the extracellular filament. However, nine other annotated PilA variants are encoded in the *Synechocystis* genome, most with unknown function (11). These PilA variants are termed “minor pilins.” The minor pilins of the *pilA9-slr2019* operon, containing *pilA9* to *pilA11*, *slr2018*, and *slr2019*, have recently been implicated in modulating the adhesive properties of T4P, switching between cell-adhesive and surface-adhesive states depending on cyclic AMP (cAMP) signaling (12). The *pilA9-slr2019* operon is regulated both on a whole-operon basis (13) and an individual gene basis for at least *pilA11* via a *cis*-encoded antisense RNA (14), controlling the number and thickness of pili. The expression of *cccP* (*slr1668*) and *cccS* (*slr1667*), two targets of the *Synechocystis* cAMP transcription factor SYCRP1, is altered in a mutant lacking the RNA chaperone Hfq (15). Hfq localizes to the pilus base and interacts with the T4P extension motor PilB1 (16). CccP and CccS have been suggested to be essential for motility, and they modulate *Synechocystis* cell surface structures and piliation (17). CccP and CccS are putative components of a *Synechocystis* chaperone-usher (CU) pilus system, with CccP as the periplasmic chaperone, CccS as a CU pilus subunit, and the product of the *slr0019* gene, UshA, as the outer membrane usher pore protein (1).

Besides motility, T4P have been shown to be involved in a range of biofilm-supporting functions. *Synechocystis* requires T4P for the uptake of extracellular DNA (18), losing its natural competency when T4P are disrupted. The presence of T4P has proven crucial both structurally (19, 20) for biofilm formation and as a source of genetic diversity via horizontal gene transfer (21, 22) in established communities. Recently, Vibrio cholerae T4P have been imaged pulling DNA to the cell surface in experiments using fluorescent probes (23). T4P also appear to be involved in sensing surfaces and signaling the production of holdfasts required for surface attachment in Caulobacter crescentus (24) and P. aeruginosa, where tension in retracting T4P is sensed and leads to gene regulation downstream (25).

When bacteria form biofilms or flocs, they secrete extracellular polymeric substances (EPS), such as polysaccharides, proteins, and DNA, to form a matrix that interconnects cells and provides structural integrity to the aggregate. Cyanobacterial EPS appears to substantially modulate the surface charge of cyanobacterial cells (26, 27) and their ability to adsorb metal ions.

EPS have been implicated in protection against stresses like high salt or metal concentrations (27) and peroxide and light stress (28) in *Synechocystis* and temperature and desiccation stress (29) in Nostoc commune, thereby providing a more constant environment for the bacteria. The benefits of EPS are reviewed in reference 30. However, EPS also limits mass transfer and the supply of nutrients in bacterial communities. In the heterotroph Bacillus subtilis, nitrogen utilization within biofilms is dynamically tuned to allow nitrogen to reach the innermost parts of biofilms (31), regulated by intercellular electrochemical signaling (32). It has been suggested that in Pseudomonas aeruginosa biofilms, genome-encoded prophages help to kill some bacteria to help shape the structure of the biofilm (33). This type of social behavior shows that bacterial aggregates are often more than the sum of their parts. Indeed, cyanobacterial flocculation seems to be an ancient-yet-conserved mechanism that made an important contribution to carbon burial in the oceans during the Paleoproterozoic (26), giving further evidence for the importance of communal lifestyles. A recent study has shown that *Synechocystis* can flocculate in response to changes in nutrient conditions (9).

Aggregation in *Synechocystis* and other cyanobacteria depends on the color of incident light, which in turn is influenced by a variety of factors, such as the position in the water column or the degree of self-shading in a culture. In cyanobacteria, a high ratio of blue to green light seems to enhance surface adsorption on glass (34, 35). Cyanobacteriochrome receptors, such as Cph2 in *Synechocystis* (36, 37) and the SesABC system in *Thermosynechococcus* (35, 38), modulate the levels of the second messenger cyclic di-GMP (c-di-GMP), producing c-di-GMP in blue light and, in the case of SesABC, breaking it down in green light. Cyclic di-GMP is a ubiquitous second messenger promoting sessility and biofilm formation in many bacterial species (39–41) and promoting cellulose-dependent aggregation in the cyanobacterium T. vulcanus (35). Cph2 in *Synechocystis* synthesizes c-di-GMP through its GGDEF (diguanylate cyclase) domain if blue light is detected by its C-terminal GAF domain (36), suggesting a possible role in aggregation in *Synechocystis*.

Biofilm formation is commonly quantified by measuring the degree of adherence of a given strain to a surface, commonly a glass test tube, via crystal violet staining. This has been applied to *Synechocystis* in previous studies (see references 34 and 42, for example). *Synechocystis* wild type (wt) commonly shows little to no adherence to the surface in such assays under standard culture conditions, while some mutations and different conditions, such as blue light, promote adherence. Fisher et al. (42) note that in plastic vessels, *Synechocystis* cells flocculate (i.e., form aggregates in the liquid phase), and indeed, slime and floc formation of *Synechocystis* liquid cultures has been reported in the past for wt cells (27) and mutants such as *pilA1* deletion mutants and polysaccharide reuptake mutants (18, 43). Because of the issues caused by the surface dependence of *Synechocystis* adherence (and the nonphysiological nature of glass surfaces), we focused our study on flocculation of *Synechocystis* rather than surface adherence.

As reported here, we devised a quantitative assay for flocculation by imaging chlorophyll autofluorescence. We applied this assay to a series of *Synechocystis* mutants with altered piliation, cell surface features, and light sensing to examine the importance of these factors for flocculation. We used fluorescence microscopy to provide insights into the internal structure of the flocs, demonstrating that flocculation is a cooperative and tightly regulated phenomenon. We showed that cells in the center of the flocs exhibit signs of nutrient stress, illustrating the physiological significance of large-scale cell organization in this cyanobacterium.

## RESULTS

### Quantifying flocculation of *Synechocystis*.

**FIG 1:**
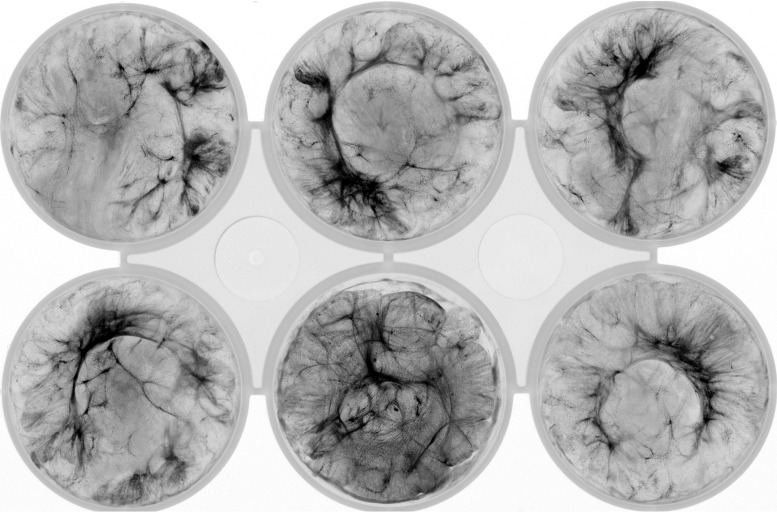
A representative flocculation assay showing wt *Synechocystis* cells after 2 days of incubation. Chlorophyll fluorescence is shown in inverted gray scale, so that the areas with highest fluorescence appear the darkest. The diameter of each well is 3.5 cm. Mean aggregation value ± standard deviation (SD) = 0.808 ± 0.088.

All our studies used the motile PCC-M strain of *Synechocystis* (44), grown as detailed in Materials and Methods. We aliquoted liquid cell cultures into plastic 6-well plates which were incubated with gentle shaking at 75 rpm, a rate chosen as the minimum that would keep nonflocculating mutants in suspension. Flocculation is prevented by rapid shaking and was not observed in the precultures shaken at 120 rpm. After 48 h of incubation, the plates were imaged in a Typhoon fluorescence imager using wavelengths appropriate for chlorophyll autofluorescence. The inhomogeneity of the fluorescence image is a measure of the degree of aggregation (Fig. 1). We quantified inhomogeneity as the standard deviation in the fluorescence image, normalized to the mean fluorescence intensity. The normalization is necessary as higher intensities have an intrinsically larger spread and, hence, a higher standard deviation. We defined the aggregation score with the equation *A* = standard deviation/mean intensity, which allows comparison between strains and conditions.

### Internal structure of flocs shows filament-like appearance.

**FIG 2:**
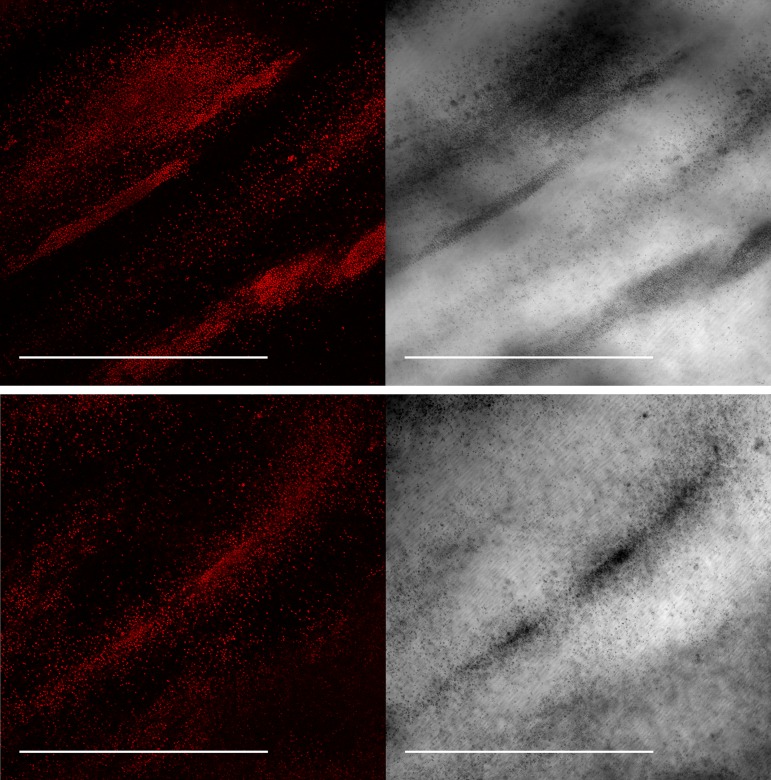
Representative confocal laser scanning microscope images of wt flocs at low magnification. *Synechocystis* chlorophyll fluorescence is shown on the left, and corresponding bright-field images are on the right. Scale bars represent 500 μm.

We probed the internal structure of flocs in solution by laser-scanning confocal microscopy. Figure 2 shows representative autofluorescence and bright-field images of wt flocs at low magnification. The flocs consist of dense areas of bacteria interspersed with regions devoid of cells that are possibly filled with EPS. The bacterial aggregates show a filamentous structure, also seen in macroscopic images (Fig. 1). The structure likely reflects patterns of active adherence of *Synechocystis* cells to each other and to the EPS, akin to the patterning observed in P. aeruginosa (7), where T4P-mediated motility is required for substructure formation.

### Flocculation of *Synechocystis* requires Hfq function.

**FIG 3:**
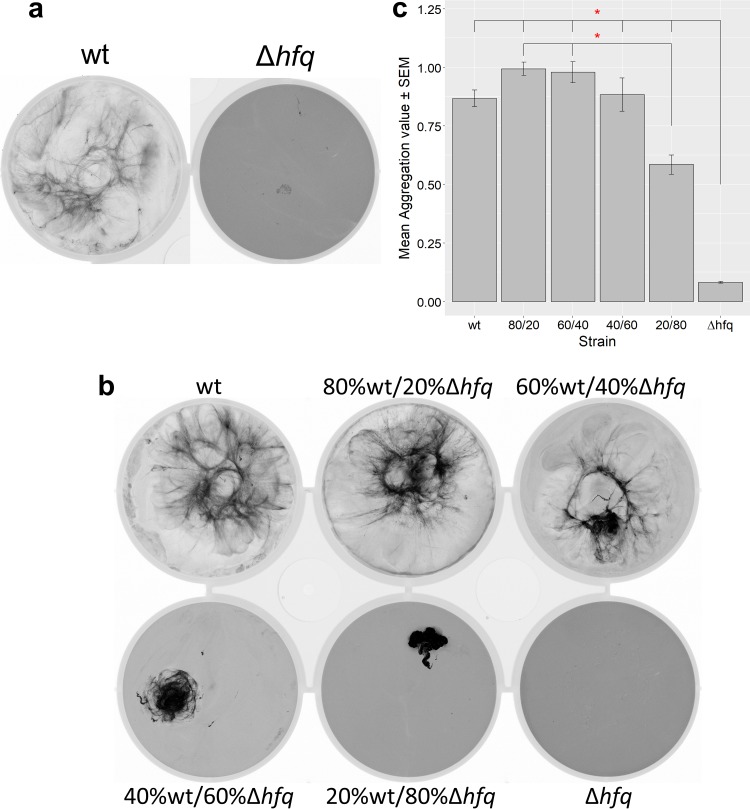
Impact of *hfq* deletion on flocculation, monitored by flocculation assays as described in the legend to Fig. 1. (a) Representative flocculation assays for wt and Δ*hfq* cultures. (b) Representative flocculation assay for cocultures of wt and Δ*hfq* cells, with cocultures started at the same density but with different ratios of the two cell types. (c) Mean aggregation values ± standard errors for wt/Δ*hfq* mutant cocultures. Asterisks indicate significance at a *P* value of <0.05.

Hfq is a putative RNA chaperone that interacts with PilB1 and is required for T4P assembly and motility in *Synechocystis*. Furthermore, Hfq controls the mRNA accumulation of a variety of genes (15, 16), including *pilA9*, *pilA10*, and *slr2018*. The cyanobacterium Synechococcus elongatus PCC 7942 apparently does not require Hfq to form biofilms (8). We therefore set out to examine the effect of Δ*hfq* mutation on flocculation in *Synechocystis*. The Δ*hfq* mutant shows perfectly evenly distributed autofluorescence, characteristic of a planktonic culture (Fig. 3a), corroborating the idea that Hfq is required for *Synechocystis* flocculation.

We next investigated whether the nonflocculating phenotype of the Δ*hfq* mutant could be rescued by coculture with the wt. We mixed cultures of wt and Δ*hfq* cells in several ratios (wt only, 80% wt/20% Δ*hfq* cells, 60% wt/40% Δ*hfq* cells, 40% wt/60% Δ*hfq* cells, 20% wt/80% Δ*hfq* cells, and Δ*hfq* cells only) to the same final *A*_750_ as in Fig. 1 and 3a and assayed for flocculation. The results of a representative flocculation assay are shown in Fig. 3b. The coculture maintains flocculation around wt levels until the percentage of Δ*hfq* cells is at 60% and above, at which point the flocs become denser and smaller, gradually losing their filamentous structure. This is reflected in the aggregation values, shown in Fig. 3c. Little autofluorescence can be detected in the medium outside the flocs in the wt wells or at 20% Δ*hfq* cells. However, higher proportions of Δ*hfq* cells result in higher background autofluorescence, indicating planktonic cells which are not incorporated into the flocs (Fig. 3b).

### GFP tagging of the Vipp1 protein gives insight into structure and stress in flocs.

**FIG 4:**
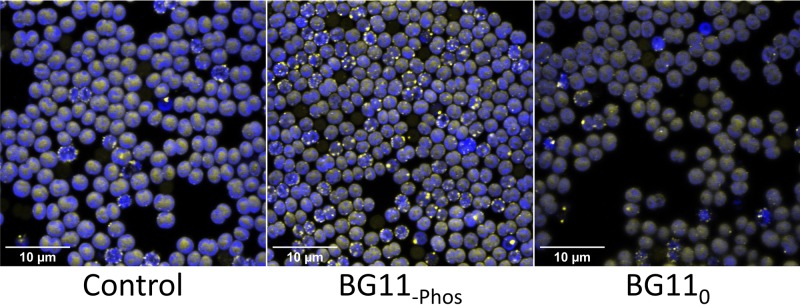
Vipp1-GFP expression and localization in planktonic *Synechocystis* cultures. Confocal fluorescence images of *Synechocystis vipp1-gfp* cells grown in planktonic culture and then spotted on an agar surface for imaging. Chlorophyll fluorescence is shown in blue, and GFP fluorescence in yellow. Overlapping areas appear white. Cells were grown for 3 days in normal BG11 medium, phosphate-free medium (BG11_-Phos_), or nitrate-free BG11_0_ medium. GFP fluorescence can appear largely diffuse (control sample) or show punctum formation (phosphate-starved sample).

Vipp1 (or IM30) is an abundant protein in *Synechocystis* that is implicated in thylakoid biogenesis, with postulated roles in stress-related protein synthesis (45) and membrane fusion (46). Green fluorescent protein (GFP) tagging of Vipp1 in *Synechocystis* revealed a stress-dependent subcellular distribution. Unstressed cells grown in low light show a dispersed signal in the cytoplasm, whereas high-light stress triggers a redistribution into sharp puncta within 30 min (45). For this study, we made a *vipp1-gfp* strain in the motile PCC-M background and verified that it shows a bright GFP signal in all cells (Fig. 4). This signal is easily distinguished by the fluorescence from the native photosynthetic pigments (45), and Förster resonance energy transfer (FRET) effects are unlikely because there is little overlap between GFP emission and photosynthetic pigment absorption. In addition to the previously known high-light effect (45), we found that nutrient stress in planktonic cultures can trigger the redistribution of Vipp1-GFP into puncta. This effect is very striking with phosphate deprivation, although nitrate deprivation has only a marginal effect (Fig. 4). We verified that the *vipp1-gfp* strain flocculates similarly to the wt (see Fig. S1 in the supplemental material), allowing us to use it as a proxy for the wt in flocculation experiments, where the Vipp1-GFP fluorescence signal can serve both as an indicator of cell type in coculture experiments and as a stress reporter.

**FIG 5:**
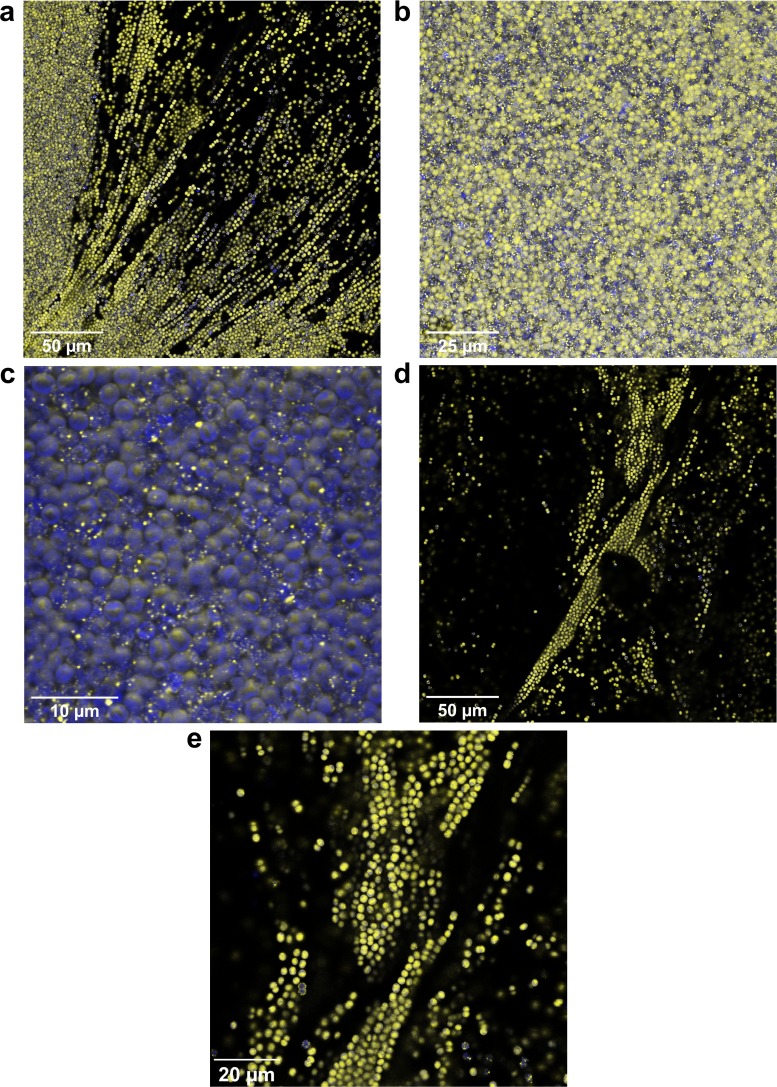
Formation of Vipp1-GFP puncta in dense regions of flocs. Confocal fluorescence micrographs with chlorophyll fluorescence shown in blue and GFP fluorescence in yellow. Overlapping areas appear white. (a) The edge of a dense part of the floc with coalescing filaments. (b) A dense area in the center of the floc with extensive formation of puncta throughout. (c) Enlarged view of part of panel b showing extensive Vipp1-GFP punctum formation. (d) An outlying region of the floc, showing a low incidence of Vipp1-GFP puncta. (e) Enlarged view of part of panel d.

*vipp1-gfp* cells were allowed to flocculate under standard conditions, transferred to glass slides, and imaged with the confocal microscope. Representative confocal micrographs are shown in Fig. 5. Vipp1-GFP is highly expressed throughout the floc, but its subcellular distribution is dependent on the cellular position within the floc, with most cells in the less dense outlying regions showing dispersed distribution (Fig. 5d and e), while formation of Vipp1-GFP puncta is prevalent in densely packed parts of the floc (Fig. 5a to c). In the outlying regions, 11% of cells showed puncta (*n* = 666), whereas punctum incidence was 50% in the denser regions of the flocs (*n* = 461). This suggests nutrient stress in cells in the denser parts of the floc.

**FIG 6:**
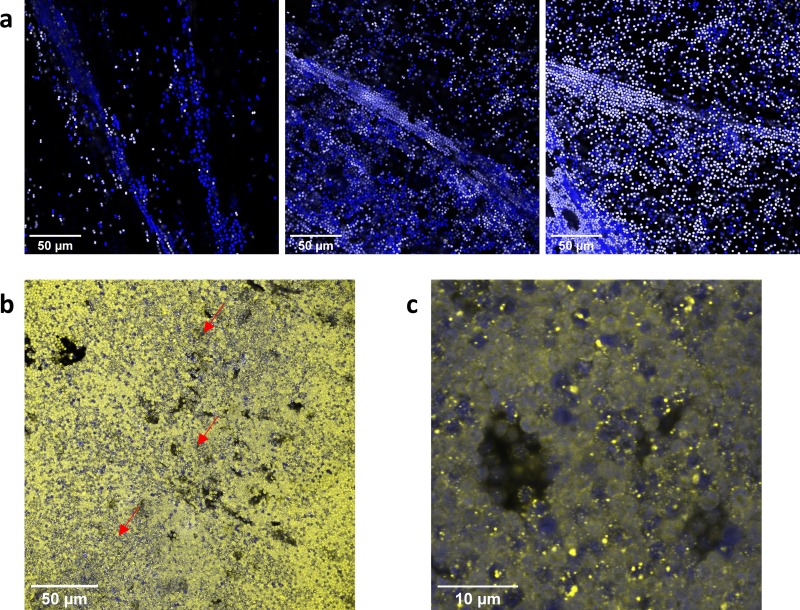
Arrangement of cells in *vipp1-gfp* and Δ*hfq* mutant coculture flocs. Confocal fluorescence micrographs with chlorophyll fluorescence shown in blue and GFP fluorescence in yellow. Δ*hfq* cells appear blue, and cells with a dispersed or punctate yellow signal are *vipp1-gfp* mutants. (a) Three areas representative of the less dense regions of the flocs. Overlapping yellow and blue signals appear white in this panel. (b) A representative dense area of the floc. Red arrows indicate areas with a high incidence of Vipp1-GFP foci. (c) Enlarged image of a dense area of the floc.

We then used *vipp1-gfp* cells as a proxy for the wt to investigate the distribution of flocculating and nonflocculating cell types in coculture experiments. All *vipp1-gfp* cells show GFP fluorescence (Fig. 4 and 5), whereas the nonflocculating Δ*hfq* cells only show weak background fluorescence, allowing us to distinguish between the two cell types. We grew coculture flocs using a protocol similar to that used for the experiment whose results are shown in Fig. 3, with a ratio of 50% *vipp1-gfp* cells/50% Δ*hfq* cells. Confocal fluorescence images are shown in Fig. 6. The coculture flocs (Fig. 6) had a filamentous structure similar to that of wt flocs (Fig. 2). Consistent with the data shown in Fig. 3, Δ*hfq* cells are incorporated into the flocs, although these cells cannot flocculate when grown in a monoculture. The less dense regions of the flocs have a mixed population of Δ*hfq* and *vipp1-gfp* cells, with frequent close association between the two cell types (Fig. 6a). In contrast, Δ*hfq* cells are very infrequent in dense regions of the flocs (Fig. 6b and c). As also seen in flocs from *vipp1-gfp* monocultures (Fig. 5), Vipp1-GFP puncta are prevalent in dense regions of the floc, suggesting nutrient stress (Fig. 6c).

### Chaperone-usher pili are not required for flocculation.

**FIG 7:**
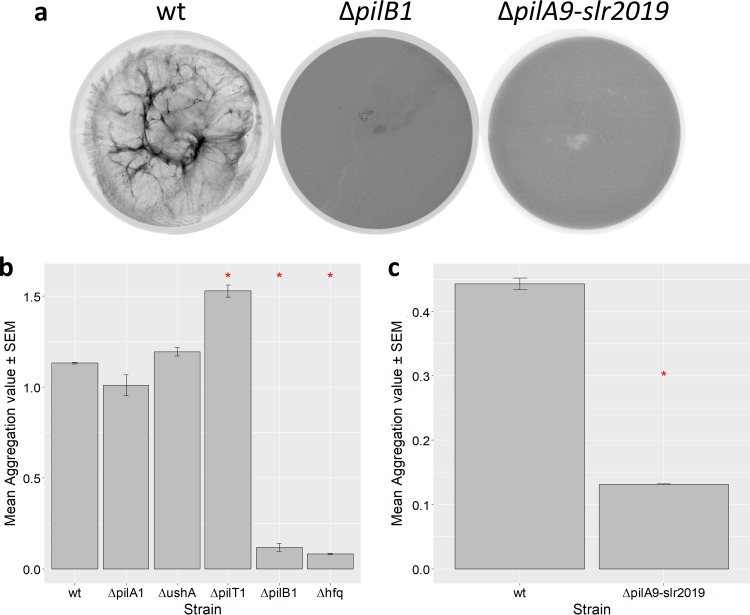
Flocculation of mutants impaired in pilus function in comparison to the flocculation of wt *Synechocystis*. (a) Representative flocculation assays (similar to those whose results are shown in Fig. 1 and 3) for Δ*pilB1* and Δ*pilA9-slr2019* mutants compared to the wt. (b) Mean aggregation scores (± standard errors) for wt and mutants lacking T4P components. Asterisks indicate significance at a *P* value of <0.05. See Table S1 in the supplemental material for data with standard deviations. (c) Mean aggregation scores (± standard errors) for wt and Δ*pilA9-slr2019* mutant. Asterisks indicate significance at a *P* value of <0.05.

There are at least two possible explanations for the nonflocculating phenotype of the Δ*hfq* mutant (Fig. 3). First, Hfq regulates the expression of *cccP* and *cccS* (15), putative components of a chaperone-usher (CU) system (1). Second, Hfq associates with PilB1 in the T4P system and is essential for T4P extension and motility (16). To test for the possible involvement of CU pili or similar surface structures, we quantified flocculation in a mutant lacking *ushA* (*slr0019*), which is the only recognizable candidate for encoding the usher pore in *Synechocystis* (1). As previously reported (12), the Δ*ushA* mutant retains motility. Δ*ushA* cells flocculated to an extent similar to the flocculation of the wt (Fig. 7), indicating that the CU system is not required for flocculation.

### Roles of T4P components in flocculation.

We investigated the role of type IV pili (T4P) in flocculation using a series of null mutants lacking specific T4P components (Fig. 7). The Δ*pilB1* mutant lacking thick T4P (18) showed no detectable flocculation, similarly to the Δ*hfq* cells. In contrast, the hyperpiliated (10) Δ*pilT1* mutant showed a significantly increased level of aggregation compared to that of the wt (Fig. 7). This shows that the amount of flocculation is influenced by the abundance of T4P but does not require active pilus retraction.

PilA1 is the major pilin component of thick pili, and *Synechocystis* Δ*pilA1* mutants are nonmotile (10). We therefore looked for an effect of *pilA1* deletion on flocculation, but surprisingly, we found that a Δ*pilA1* mutant shows no significant difference in flocculation compared to the wt (Fig. 7). This indicates that neither T4P composed of the major pilin PilA1 nor twitching motility is required for flocculation.

The mutant phenotypes (Fig. 7) leave adhesion mediated by appendages composed of one or more minor pilins as the best remaining candidate for the factor required to link the cells in the flocs. We therefore tested for flocculation in a mutant lacking the operon encoding the minor pilins PilA9-slr2019. In contrast to the Δ*pilA1* mutant, the Δ*pilA9-slr2019* mutant appears strongly deficient in flocculation (Fig. 7). This implies that at least one of these minor pilins is crucial for cell-cell adhesion in flocculation.

### Cph2 promotes flocculation depending on the wavelength of incident light.

**FIG 8:**
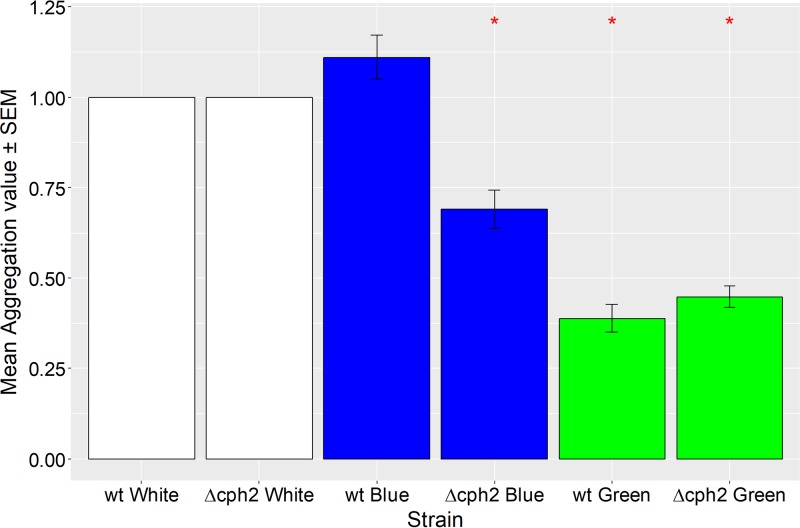
Flocculation of *Synechocystis* wt and Δ*cph2* cells depending on the color of illumination. Mean aggregation values ± standard errors are shown for white, blue, and green light-illuminated wt and Δ*cph2* cells. Data are normalized to the values for white light controls. Asterisks indicate significant differences from the results for the blue light-illuminated wt (*P* < 0.05).

In *Synechocystis*, the cyanobacteriochrome Cph2 detects and transduces light signals by synthesizing the second messenger cyclic di-GMP in response to blue light (36). We used a Δ*cph2* mutant (47) to investigate the possible role of this photoreceptor in the control of flocculation. We carried out flocculation assays for wt and Δ*cph2* cultures in white, green, or blue light (Fig. 8). Data were normalized to the results for wt or Δ*cph2* culture white light controls. There was no significant difference between the wt and the *Δcph2* mutant in white light before normalization (Fig. S2). Wild-type cultures flocculate to similar extents in white and blue light but much less in green light. Δ*cph2* cells show relatively less flocculation in blue light, and the difference in flocculation between blue and green light is greatly reduced in comparison to the results for the wt (Fig. 8). Wild-type and Δ*cph2* cells show no difference in green light. The difference between blue and green light-illuminated samples is in keeping with data on surface adherence in *Synechocystis* (34) and Thermosynechococcus vulcanus (35). The results with Δ*cph2* suggest that blue light-induced c-di-GMP production is a major factor in promoting flocculation in *Synechocystis*.

### Bicarbonate addition decreases flocculation of *Synechocystis*.

**FIG 9:**
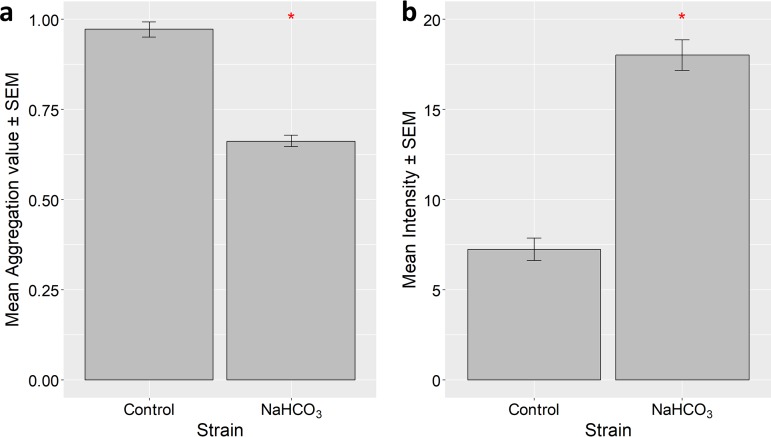
Impact of 10 mM extracellular NaHCO_3_ on flocculation of *Synechocystis*. (a) Mean aggregation value ± standard error for wt *Synechocystis* with or without 10 mM extracellular NaHCO_3_ added. (b) Mean autofluorescence intensities for the same readings. Asterisks indicate significant difference at a *P* value of <0.05.

Kamennaya et al. recently showed that the marine species *Synechococcus* sp. strain PCC 8806 shows approximately linearly increasing flocculation as CO_2_ availability increases (26). We therefore tested the effect of boosting CO_2_ availability by supplementing *Synechocystis* cultures with 10 mM NaHCO_3_ in our flocculation assay (Fig. 9). Bicarbonate addition resulted in a significantly lower aggregation score (Fig. 9a) and significantly boosted cell growth during incubation (Fig. 9b). Thus, there is no indication that higher CO_2_ availability promotes flocculation in *Synechocystis*. The lower aggregation score in the bicarbonate-supplemented culture might be explained by self-shading in the denser culture, leading to a lower ratio of blue/green light (Fig. 8).

## DISCUSSION

Our results shed light on the phenomenon of flocculation in the model cyanobacterium *Synechocystis*. Slime formation in the bottom of *Synechocystis* culture flasks, as well as flocculation of *Synechocystis*, have been mentioned in previous studies (27, 42, 43). Flocculation is clearly related to biofilm formation but depends on direct or indirect cell-cell adhesion, rather than a combination of cell-cell and cell surface adhesion. Natural phototrophic microbial mats are commonly stratified into layers, with cyanobacteria residing near the surface of the mat (48). Therefore, in microbial mats and similar communities, most interactions will be between cyanobacteria and other cells, rather than between cyanobacteria and the substratum. In contrast to another recent study on biofilm formation and aggregation in *Synechocystis* (9), we found that wt *Synechocystis* readily forms flocs when grown in standard BG11 medium, without any requirement for environmental perturbation, such as nutrient deprivation. The difference may be due to the properties of the substrain of wt *Synechocystis* used: we employed the highly motile PCC-M substrain (44).

To investigate the factors that promote flocculation, we developed a quantitative flocculation assay. Our assay requires no additional staining, instead relying on *Synechocystis* autofluorescence only. It enables the quantitation of phenotypes that show less-than-wt flocculation. This is an advantage over established methods for quantifying surface adherence and cell aggregation, as *Synechocystis* wt cells show very little surface adherence in crystal violet adhesion assays (34, 42).

Microscopic investigation of the flocs formed in our assays shows that they consist of chains of cells at the periphery, interspersed with cell-free voids. In dense regions of the flocs, the cell strands pack together to form a dense mass of cells interspersed with occasional small cavities (Fig. 2 and 5). We suggest that the voids between the dense filaments in *Synechocystis* flocs are analogous to B. subtilis biofilm wrinkles, which have been shown to form channels through the biofilm that substantially enhance mass transfer (49). The small-scale cavities found in some of the densest areas of the *Synechocystis* flocs (Fig. 5) likely serve the same purpose on a smaller scale. They could allow CO_2_ and nutrients to freely enter the floc and diffuse within it, while facilitating the dissipation of high O_2_ concentrations found in phototrophic layers of bacterial mats (48) and even small flocs (26). The filamentous structure of the flocs might enable better nutrient distribution into denser regions. Nevertheless, we found that cells in the denser center regions of the flocs show signs of stress, as judged from the subcellular distribution of Vipp1-GFP, a cytoplasmic protein which coalesces into puncta under stress conditions (45). Punctum formation can be induced by high-light stress (45) or nutrient starvation (Fig. 4). For our assays, cells were grown in relatively low light intensity, and therefore, punctum formation was most probably induced by nutrient stress. Phosphate deprivation can act as a strong trigger for Vipp1-GFP punctum formation (Fig. 4), but an influence of the levels of other nutrients cannot be excluded.

Flocculation assays in which wt (or *vipp1-gfp*) *Synechocystis* cells are mixed with cells of the nonflocculating Δ*hfq* mutant show that flocculation is a rather cooperative phenomenon. Δ*hfq* cells are readily incorporated into flocs in the looser filamentous regions but tend to be excluded from the densely packed regions (Fig. 6). At proportions of Δ*hfq* cells of about 60% or above, the floc structure is strongly disrupted, with loss of the loose filamentous regions and an increasing background of planktonic cells (Fig. 3). The formation of loose cell strands presumably depends on having a sufficient proportion of cells that actively contribute to cell-cell adhesion: these strands appear to be destabilized by the incorporation of too many cells with the nonflocculating phenotype.

Our results with mutants perturbed in a variety of cell surface features show that *Synechocystis* flocculation requires only a very specific subset of surface components. Pili of the chaperone/usher system (1) appear to play no role in flocculation, since loss of UshA, the essential outer membrane pore of the CU system, has no effect (Fig. 7). A recent study implicated a role of T4P in *Synechocystis* flocculation, since loss of PilC, the T4P inner membrane platform, prevented flocculation (9). Our results confirm the involvement of T4P and provide more detail on the specific aspects of T4P function that are important for flocculation. Δ*pilB1* cells, which are unable to assemble T4P, do not flocculate (Fig. 7). Δ*pilT1* cells, which are hyperpiliated because they can extend T4P but not retract them, show a significantly higher level of aggregation than the wt (Fig. 7). This indicates that cell adhesion for flocculation depends on T4P but does not require the active cycles of pilus extension and retraction that are involved in motility. PilA1 is quantitatively the major subunit of T4P and is essential for motility (10). Remarkably, however, loss of PilA1 had little or no effect on flocculation, whereas flocculation was strongly deceased in a mutant lacking the operon coding for the minor pilins PilA9 to PilA11, slr2018, and slr2019 (Fig. 7). The result implies that a specific subset of T4P components assembled from one or more of these minor pilins is responsible for cell-cell adhesion in flocculation. The flocculation pili must require the standard T4P components (including the PilB1 extension motor and the PilC inner membrane platform) for their assembly but may lack PilA1 completely. Δ*pilA1* mutants lack the “thick” T4P that can be observed by electron microscopy but retain thinner pili (10) that presumably include pili assembled from minor pilin subunits. It is an open question whether such pili are retractile or not, but the enhancement of flocculation in the Δ*pilT1* cells suggests this as a possibility. The possible role of the second *Synechocystis* PilT homolog, PilT2 (10), also remains to be investigated.

We were able to identify light quality as one factor that regulates flocculation in *Synechocystis*. Green light significantly inhibits flocculation relative to the effect of blue light, and this effect is greatly decreased (but not completely abolished) in a mutant lacking the photoreceptor Cph2 (Fig. 8). The C-terminal domain of Cph2 is a known sensor of the blue/green light ratio and produces the second messenger c-di-GMP upon activation by blue light (36). c-di-GMP is therefore likely to promote flocculation, in addition to its previously established effect in inhibiting motility (36). The small remaining blue/green difference in the response seen in Δ*cph2* cells (Fig. 8) may indicate the influence of another blue light-regulated signaling pathway. One candidate is cyclic AMP (cAMP), which is generated in blue light illumination (50). This would be consistent with a recent suggestion on the impact of cyclic AMP on *Synechocystis* surface features (12). The enhanced flocculation at higher blue/green light ratios could allow Synechocystis to tightly regulate the light environment that flocculating cells experience by promoting flocculation in planktonic cultures but at the same time limiting excessive flocculation that can lead to self-shading and light deprivation in the center of the floc. This a likely explanation for the lower flocculation that we observed in the denser cultures induced by NaHCO_3_ supplementation of the medium (Fig. 9).

Our results indicate that flocculation is a highly specific process, dependent on a specific subset of minor pilins and subject to complex regulation. This implies that it must confer an evolutionary advantage, despite the signs of nutrient stress that we observed in cells in the dense regions of the flocs (Fig. 5). However, the physiological benefits of flocculation remain to be established. Aggregation has been suggested to be a mechanism to avoid photodamage by self-shading in T. vulcanus (35), and other possible benefits for cyanobacterial cells in the wild could include protection from predation, control of buoyancy in the water column, and the establishment of mutually beneficial microbial communities, perhaps including heterotrophs as well as cyanobacteria. Better knowledge of the behavior and interactions of *Synechocystis* in its natural environment will be essential if we are to understand the reasons for complex phenomena like flocculation and motility.

## MATERIALS AND METHODS

### Culture growth conditions.

The motile *Synechocystis* strain used in this study is PCC-M (44). Construction of the Δ*hfq* and Δ*cph2* mutant strains was described previously, in references 15 and 47, respectively. *Synechocystis* cultures were grown in BG11 medium (51) buffered with [Tris(hydroxymethyl)methyl]-3-aminopropanesulfonic acid (TAPS) to pH 8.2 in plastic tissue culture flasks (Sarstedt), incubated at 30°C under continuous illumination (15 μmol photons m^−2^ s^−1^) and with agitation (120 rpm). Strains were maintained on plates containing appropriate antibiotics, but liquid cultures were grown without antibiotics to prevent distorting effects.

### Mutagenesis.

The mutants used are listed in Table S2 in the supplemental material. Deletion mutants with mutations in *pilA1* (*sll1694*), *pilB1* (*slr0063*), and *pilT1* (*slr0161*) were generated by transformation with pGEM-T easy-based constructs (Δ*pilA1*, Δ*pilB1*, and Δ*pilT1* deletion constructs). The constructs contained a resistance gene for chloramphenicol (Δ*pilA1*), a resistance cassette for kanamycin (Δ*pilB1*), or a resistance gene for apramycin (Δ*pilT1*), each flanked by genomic upstream and downstream sequences of the gene of interest containing overhangs to allow for later assembly. The upstream/downstream sequences were 1,000 bp long for all constructs. The primers used are shown in Table S3. The flanking sequences for the pGEM-T easy-Δ*pilA1*-CamR construct were amplified from the *Synechocystis* genome using primer pairs US-A-pilA1 KO/US-S-pilA1 KO (upstream) and DS-A-pilA1 KO/DS-S-pilA1 KO (downstream). The flanking sequences for the pGEM-T easy-Δ*pilB1*-KanR construct were amplified from the *Synechocystis* genome using primer pairs US-A_PilB1 KO/US-S_PilB1 KO (upstream) and DS-A_PilB1 KO/DS-S_PilB1 KO (downstream). The flanking sequences for the pGEM-T easy-Δ*pilT1*-ApraR construct were amplified from the *Synechocystis* genome using primer pairs US_A_PilT KO/US_S_PilT KO (upstream) and DS_A_PilT KO/DS_A_PilT KO (downstream). Colony PCRs were performed using Q5 polymerase (New England Biolabs, USA). Antibiotic resistance genes were amplified using primer pairs CmR-A-pilA1 KO/CmR-S-pilA1 KO (Cm^r^ Δ*pilA1* construct), KmR-A_PilB1 KO/KmR-S_PilB1 KO (Km^r^ Δ*pilB1* construct), and AprR_A_PilT KO/AprR_S_PilT KO (Am^r^ Δ*pilT1* construct). The PCR-generated overhangs were then used to assemble the construct using NEBuilder HiFi DNA assembly 2× master mix (NEB, USA), following the manufacturer’s instructions. The constructs’ sequences were confirmed by sequencing.

For inactivation of *ushA* (*slr0019*), the peripheral regions of the gene were amplified by primer pairs slr019-A-fw/-rev and slr0019-B-fw/-rev, subsequently fused by overlap extension PCR using the outward primers, and ligated into a pJET1.2 cloning vector. A kanamycin resistance gene from plasmid pUC4K was excised by EcoRI digestion and inserted into the corresponding site between the flanking *slr0019* sequences. After transformation of *Synechocystis*, complete genomic segregation was verified by PCR analyses.

For deletion of the *pilA9-slr2019* transcriptional unit, two DNA fragments (1,190 and 1,121 bp) were amplified from the genomic DNA of *Synechocystis* wt upstream and downstream from the *pilA9* and *slr2019* open reading frames by PCR using the primer pairs P7 and P8 or P9 and P10, respectively. Both fragments were further fused by a second PCR using P7 and P10 and ligated into pJET1.2, thereby removing the complete *pilA9-slr2018* and half of the *slr2019* sequence. NdeI and SphI recognition sequences were introduced through the primers. A kanamycin resistance gene cassette was amplified by PCR from the pUC4K vector (52) using P11 and P12 and ligated using NdeI and SphI restriction enzyme recognition sites introduced into the pJET vector. Complete segregation of the mutant copies was checked by PCR using primers P13 and P14.

The Vipp1-GFP construct was generated in accordance with the method of Bryan et al. (45) using a pRL271-based vector.

*Synechocystis* cells were transformed, and segregation was confirmed by PCR. The final genomic alterations are summarized in Fig. S3.

Transformations were performed by adding 2.5 μg construct to 500 μl *Synechocystis* wt cells at an *A*_750_ of 0.8, incubating the mixture overnight before plating on BG11 agar plates, incubating the plates for 2 days before the addition of half-strength antibiotics (kanamycin, apramycin, and spectinomycin, 25 μg/ml final concentration, and chloramphenicol, 12.5 μg/ml final concentration) underneath the agar plate, and incubating further until colonies appeared.

### Aggregation assays.

Aggregation assays were performed by diluting exponential-phase *Synechocystis* cultures to an *A*_750_ of 0.5 (Jenway 6300 spectrophotometer), roughly equivalent to 2.25 μM chlorophyll *a* (Chl *a*), in Corning Costar TC-treated 6-well plates. Each well was filled with 5 ml of culture. The assays were sealed and incubated for 48 h at 30°C, with 30 μmol photons m^−2^ s^−1^ and shaking at 75 rpm (16-mm orbit diameter/2-mm stroke in SI50 orbital incubator; Stuart Scientific, UK). After incubation, the plates were imaged on a Typhoon Trio imager (GE, USA), using the 488-nm laser line for excitation and the 670-nm BP30 emission filter to capture *Synechocystis* autofluorescence. The photomultiplier tube (PMT) voltage was set to 350 V, and the focal plane was +3 mm. The pixel size used was either 25 μm or 50 μm. Images were analyzed using ImageQuant TL (GE, USA), which allowed area selection for each well and provided a readout of the mean intensity and the standard deviation of intensities that were used to compute the aggregation value for each well. Statistical analysis was performed using one-way analyses of variance (ANOVAs) in R 3.5.1.

For light color experiments, colored filters numbers 716 and 139 (Lee Filters, UK) were used to make blue and green sleeves, respectively, enveloping the 6-well plates. The blue sleeves have their transmission maximum at 445 nm with minimal transmission in the green wavelengths, while the green sleeves have their transmission maximum at 525 nm with minimal transmission in the blue wavelengths. The light intensity used in these experiments was 70 μmol photons m^−2^ s^−1^ outside the sleeves.

For experiments involving extracellular NaHCO_3_, aggregation assays were set up as described above, with 50 μl sterile 1 M NaHCO_3_ added to one set of wells and 50 μl sterile double-distilled water (ddH_2_O) to the control wells. Assays were then run as described above. Data were analyzed using the Wilcoxon signed-rank test in R 3.5.1.

### Confocal microscopy.

Flocs were prepared by incubating 2 ml of *Synechocystis* culture (*A*_750_ = 0.5) in a Nunc Lab-Tek 2-well chamber slide for 48 h at 30°C, with 30 μmol photons m^−2^ s^−1^ and shaking at 75 rpm (SI50 orbital incubator; Stuart Scientific, UK) for samples imaged directly (Fig. 2). Samples used in the experiments whose results are shown in Fig. 4 and 6 were grown as for a regular aggregation assay (see above) and imaged between cover slip and slide. Samples were transferred to a Leica TCS-SP5 laser scanning confocal microscope equipped with a ×10 magnification air objective with a numerical aperture of 0.4 (Fig. 2) or a ×63 magnification oil immersion objective with a numerical aperture of 1.4 (Fig. 4 and 6). Cellular Chl fluorescence was imaged by excitation with the 488-nm laser line of an argon laser using a 670-nm to 720-nm emission range. GFP fluorescence was imaged by excitation with the 488-nm laser line and emission measured at 500 nm to 520 nm. Images were analyzed using the Leica LAS AF software (Leica, Germany) and the Bio-Formats plugin (53) of the Fiji distribution (54) of ImageJ.

## Supplementary Material

Supplemental file 1
