# Supplementary material for: Factors Controlling Floc Formation and Structure in the Cyanobacterium Synechocystis sp. Strain PCC 6803
Source: J Bacteriol. 2019 Sep 6;201(19):e00344-19. doi: 10.1128/JB.00344-19 (PMC6755745; doi:10.1128/JB.00344-19)
Supplement: Supplemental file 1 [file JB.00344-19-s0001.pdf]

Factors controlling floc formation and structure in the cyanobacterium *Synechocystis* sp. PCC6803

Fabian D Conradi, Rui-Qian Zhou, Sabrina Oeser, Nils Schuergers, Annegret Wilde and Conrad W Mullineaux

Supplemental Material

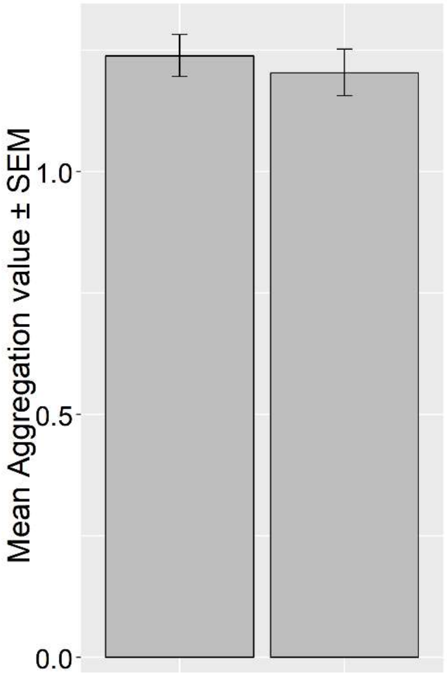

Supplementary Figure 1. **Comparison of aggregation values of wt and *vipp1-gfp* mutant.**

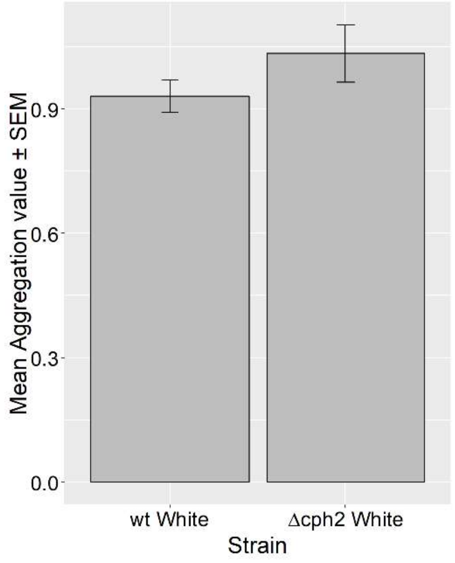

Supplementary Figure 2. **Aggregation values of wt and Δ*cph2* cells in white light before normalization.**

Supplementary Fig. 3

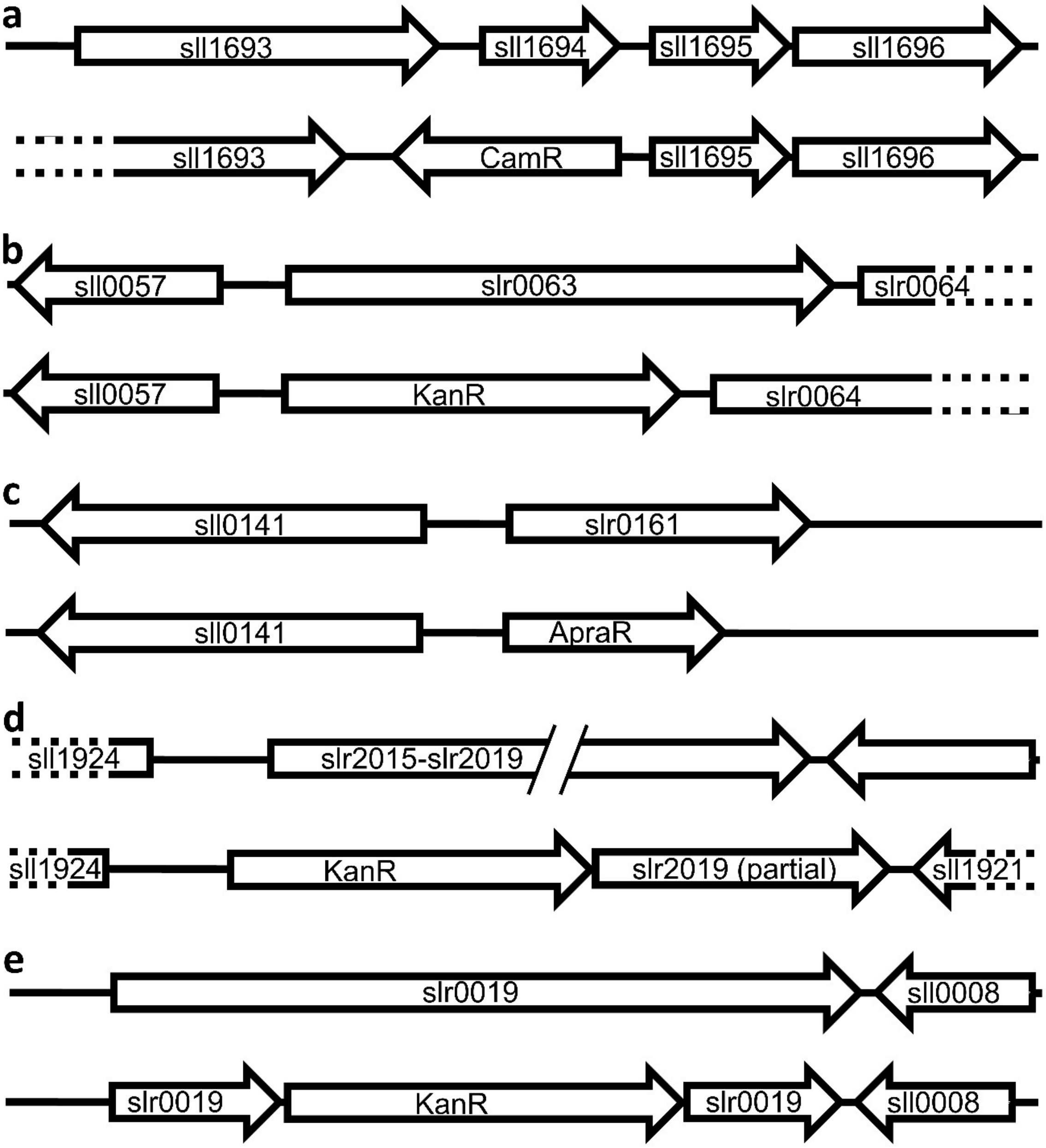

Supplementary Figure 3. **Changes in the *Synechocystis* genome for mutants produced in this study showing the original sequence (top) and the modified sequence (bottom). 1cm is equivalent to ca. 250bp. a** shows Δ*pilA1.* **b** shows Δ*pilB1*. **c** shows Δ*pilT1.* **d** shows Δ*pilA9-slr2019*. **e** shows Δ*ushA*.

Supplementary Table 1

Strain	Aggregation value	Standard deviation
wt	1.166	0.280
Δ*pilA1*	1.062	0.464
Δ*ushA*	1.194	0.134
Δ*pilT1*	1.603	0.459
Δ*pilB1*	0.118	0.065
Δ*hfq*	0.082	0.011

Mean aggregation values with standard deviations for the data shown in Fig. 7b.

Supplementary Table 2

**Mutant**	**Source**
Δ*hfq*	Dienst et al. 2008 (1)
Δ*cph2*	Wilde et al., 2002 (2)
Δ*pilB1*	This study
Δ*pilT1*	This study
Δ*pilA1*	This study
Δ*pilA9-slr2019*	This study
Δ*ushA*	This study
Vipp1-GFP	This study

Supplementary Table 2. **Source of mutants used in this work.**

Supplementary Table 3

Primer name	***Sequence (5'->3')***
	ΔpilA1
US-A-pilA1 KO	*CAGGGCGGGGCGTAAGACCCTATTATGTTTTGAGT*
US-S--pilA1 KO	*TCACTATAGGGCGAACAAAAATAGAAGTGCTAGTT*
CmR-A-pilA1 KO	*GGAAGAAGACAATCAATGCCATGGAGAGTAAAATC*
CmR-S-pilA1 KO	*AAACATAATAGGGTCTTACGCCCCGCCCTGCCACT*
DS-A-pilA1 KO	*ATAGAATACTCAAGCATGGCATTATTCGGGGTAAT*
DS-S-pilA1 KO	*TACTCTCCATGGCATTGATTGTCTTCTTCCTTCTG*
pGEM-A-pilA1 KO	*GCACTTCTATTTTTGTTCGCCCTATAGTGAGTCGT*
pGEM-S-pilA1 KO	*CCCGAATAATGCCATGCTTGAGTATTCTATAGTGT*
	ΔpilB1
US-A_PilB1 KO	*GTAATACTCGTCAACAGCAAGACCTACCCAGATTC*
US-S_PilB1 KO	*TCACTATAGGGCGAAGGACAGTGGAATGTCCCCCA*
KmR-A_PilB1 KO	*CACCCCCATCGCTATTTAGAAAAACTCATCGAGCA*
KmR-S_PilB1 KO	*TGGGTAGGTCTTGCTGTTGACGAGTATTACCCGGC*
DS-A_PilB1 KO	*ATAGAATACTCAAGCGTGGTTCAATGTCGGCAAAA*
DS-S_PilB1 KO	*GATGAGTTTTTCTAAATAGCGATGGGGGTGAGGGG*
pGEM-A_PilB1 KO	*GACATTCCACTGTCCTTCGCCCTATAGTGAGTCGT*
pGEM-S_PilB1 KO	*CCGACATTGAACCACGCTTGAGTATTCTATAGTGT*
	ΔpilT1
US_A_PilT KO	*CACCGCTGATGACATGACTTTAATGCTCCTATAAGTTCTG*
US_S_PilT KO	*TCACTATAGGGCGAACTGCCTGTTCCGCCTGTTGGGCACC*
AprR_A_PilT KO	*GTATATTTCCGTATGTCAGCCAATCGACTGGCGAGCGGC*

AprR_S_PilT KO	*AGGAGCATTAAAGTCATGTCATCAGCGGTGGAGTGCAATG*
DS_A_PilT KO	*ATAGAATACTCAAGCCACAGTATTACAAACAATTTCCAAA*
DS_S_PilT	*CAGTCGATTGGCTGACATACGGAAATATACCGATTAACAT*
pGEM_A_PilT	*AGGCGGAACAGGCAGTTCGCCCTATAGTGAGTCGTATTAC*
pGEM_S_PilT KO	*GTTTGTAATACTGTGGCTTGAGTATTCTATAGTGTCACCT*
	ΔushA (slr0019)
slr019-A-fw	*CGATTCTGCCTTGGATTTGT*
slr0019-A-rev	*CATCAGCATAAACGAATTCGACAATGGTTTTGCGGAGAT*
slr0019-B-fw	*CAAAACCATTGTCGAATTCGTTTATGCTGATGGGCGTTT*
slr0019-B-rev	*GGGGAGGATCACCGAATAAT*
	ΔpilA9-slr2019
P7 / *US763-fw*	*TCAGATCGAAGTGGGATTGC*
P8 / *NdeI-US763-rev*	*GCATGCTACATATGGGTCGATTGGGGTTTTTG*
P9 / *SphI-DS763-fw*	*CATATGTAGCATGCCTATCGGGACAGTAATC*
P10 / *DS763-rev*	*CATTGGGTAAGGGTAAGGATTTC*
P11 / *NdeI-KmR-fw*	*GCCATATGTTGTGTCTCAAAATCTCTGATG*
P12 / *KmR-fw-SphI*	*TAGCATGCTGAGGTCTGCCTCGTG*
P13 / *TU-763-Col-fw*	*CACGGTCTTTGCTGACTTC*
P14 / *TU-763-Col-rev*	*CCCGTTGATTACTGTCCC*

Supplementary Table 3. **Primers used in mutagenesis in this work.**

References

1. Dienst D, Dühring U, Mollenkopf HJ, Vogel J, Golecki J, Hess WR, Wilde A. 2008. The cyanobacterial homologue of the RNA chaperone Hfq is essential for motility of *Synechocystis* sp. PCC 6803. Microbiology 154:3134–3143.
2. Wilde A, Fiedler B, Börner T. 2002. The cyanobacterial phytochrome Cph2 inhibits phototaxis towards blue light 44:981–988.
